# Reducing trailing limb collisions in older adults through targeted leading limb placement after obstacle crossing: effect of closer foot placement

**DOI:** 10.3389/fspor.2025.1528075

**Published:** 2025-01-27

**Authors:** Tomoki Hakamata, Takahiro Higuchi

**Affiliations:** ^1^ Department of Health Promotion Science, Tokyo Metropolitan University, Tokyo, Japan; ^2^ Department of Rehabilitation, Kasai Central Hospital, Tokyo, Japan

**Keywords:** older adults, walking, obstacle crossing, collision avoidance, motion analysis

## Abstract

**Introduction:**

Older adults experience a higher frequency of collisions with obstacles when stepping over obstacles, particularly with the trailing limb. We recently demonstrated that placing the leading limb closer to an obstacle after crossing effectively increases the toe height of the trailing limb, resulting in reduced collision. This study investigated whether the intervention of placing the leading limb closer to the obstacle is effective in reducing trailing limb collisions in healthy older adults.

**Methods:**

Twenty-one older individuals (11 males, 10 females; mean age 75.7 years) participated. Participants were allocated to one of two groups: a closer placement group, instructed to place the leading limb on a square target positioned on their walking path after crossing an obstacle, and a control group, instructed to cross the obstacle naturally. The target in the closer group was set at 0.5 times the leading limb's foot placement distance, as determined in a pre-test for each participant. The experimental design included a pre-test, intervention, and post-test.

**Results and discussion:**

The collision rate for the trailing limb in the closer group was significantly lower in the post-test than that in the pre-test. Furthermore, the variability in toe height and walking speed of the trailing limb in the closer group decreased significantly in the post-test compared to the pre-test. These findings suggest that the intervention of placing the leading limb foot closer to the obstacle after crossing may improve obstacle avoidance by the trailing limb in healthy older adults.

## Introduction

Older adults experience obstacle-related collisions more frequently than younger adults when stepping over obstacles of a certain height (1), increasing their risk of falls (2–4). In particular, older adults typically demonstrate lower foot clearance step over obstacles, especially with the trailing limb (1, 5), and this clearance tends to be more variable (6–10). Given that these age-related changes increase the risk of tripping (9, 10), interventions aimed at reducing the incidence of obstacle collisions in older adults are necessary.

Following a tripping event, preventing falls requires adequate control over the forward shift of the body's center of gravity to maintain balance. Common strategies to recover balance include applying force to the ground with the supporting limb and/or executing a recovery step with the swing limb (11, 12). However, numerous studies have shown that aging may impair the ability to employ such strategies. For example, the peak ankle joint moment in the supporting limb is reduced in older adults (12), and the timing of muscle activation for the hip flexors and knee extensors is delayed when executing a rapid stepping strategy with the swing limb (13). Additionally, step reaction times also tend to be delayed in older adults (14). Given these age-associated limitations, the most effective approach for preventing a fall is to lift the foot sufficiently and avoid tripping altogether.

Recently, we demonstrated that placing the leading limb close to the obstacle after stepping over it effectively increased the toe height of the trailing limb, thereby reducing collisions (15). The intervention targeted trailing limb collision avoidance, as collisions involving the trailing limb occurred more commonly than collisions involving the leading limb (16–18). We conducted this experiment in a virtual reality (VR) environment so that physical collisions, which could dramatically change behavior (16) and hinder the effect of experimental manipulation to improve behavior, did not occur. In the study by Hakamata et al. (15), placing the leading limb close to the obstacle after crossing may cause the stance limb to be placed slightly further away from the obstacle, creating adequate space to avoid collisions of the trailing limb. Further, placing the leading limb close to the obstacle could also lead to careful control over limb movement due to a speed-accuracy trade-off. Notably, Chou and Draganich have already examined the effect of altering foot placement on contact rates (19). The difference between their study and Hakamata et al. is that, while Chou and Draganich altered the trail foot placement before the obstacle, Hakamata et al. altered the lead limb placement after the obstacle. This change was based on the suggestion from a previous study indicating that the trailing limb may have a lower priority for cognitive information processing when planning movement (20), possibly because the leading limb has a higher risk of causing a fall when it collides with an obstacle compared to the trailing limb (16). We therefore considered that altering the foot placement of the leading limb could avoid the risk of reduced control priority for the leading limb, as well as achieving similar benefits to modifying the foot placement of the trailing limb.

In that study, we compared three specified conditions for foot placement of the leading limb after stepping over the obstacle: a position relatively close to the obstacle (closer condition), a naturally selected position (middle condition), and a position relatively far from the obstacle (farther condition). A fourth condition allowed participants to choose foot placement freely without specification (control condition). The results showed that the trailing limb collision rate was lower for the closer condition than for the other three conditions. The results also showed that in the closer placement, the trailing limb passing speed decreased, suggesting greater careful control over limb movement. These findings suggest that, at least in young individuals, placing the leading limb closer to the obstacle after crossing may facilitate safe obstacle avoidance by the trailing limb.

The present study examined the effectiveness of this intervention in healthy older adults. In contrast to Hakamata et al. (15), this study was conducted in a real environment to avoid the potential risks of imbalance associated with walking while wearing a relatively heavy head-mounted display and to avoid VR sickness. Participants were divided into two groups: a closer group and a control group. This grouping was informed by previous findings, where only the closer condition demonstrated a reduction in trailing limb collisions (15), whereas the middle and farther conditions did not. The control group was included for direct comparison with the closer group. We hypothesized that an intervention involving closer foot placement of the leading limb relative to the obstacle would effectively reduce trailing limb collisions. We also hypothesized that by careful control of limb movement, indicated by slower passing speeds of the trailing limb, increased toe height, and reduced variability in toe height, would be observed. As explained, Chou and Draganich (19) have already examined the effect of altering foot placement on contact rates. The strengths of our current study, in comparison to their study, are the inclusion of a control group and the shorter duration required for the intervention.

## Materials and methods

### Participants

Twenty-one older individuals (11 males and 10 females, mean age = 75.7 years, SD = 5.7 years) participated in the study. The sample size was determined based on the data of the trailing limb collision rate reported in a previous study (15). The sample size was calculated *a priori*, requiring a total of 16 participants to detect a within-between interaction in a mixed repeated-measures design with *α* = 0.05, power (1 − β) of at least 0.80, and an effect size of *f* = 0.4 (GPower Version 3.1.9.2, Germany) (21). Participants were recruited from a mailing list of adults aged 65 years and older. To ensure eligibility, all participants were screened for normal or corrected-to-normal vision, absence of musculoskeletal injuries, and absence of neurological disorders. Cognitive and mobility functions were assessed using the Mini Mental State Examination [MMSE (22)] and the Timed Up and Go (TUG) test (23). Inclusion criteria included no cognitive impairment [MMSE score ≥24 points; (22)] and no mobility impairment [TUG score <13.5 s (24)]. The mean standing height was 162.2 cm (SD = 10.6 cm), and the mean limb length was 80.2 cm (SD = 4.6). All participants demonstrated right-limb dominance, defined as the limb used for kicking (25). The study was approved by the Ethics Committee of Tokyo Metropolitan University, Japan (Approval No.: H5-150). Written informed consent was obtained from all participants in accordance with the Ethics Committee of the Tokyo Metropolitan University and the Declaration of Helsinki. Participants received a bookstore gift card as a reward for their participation.

### Apparatus

**Figure 1:**
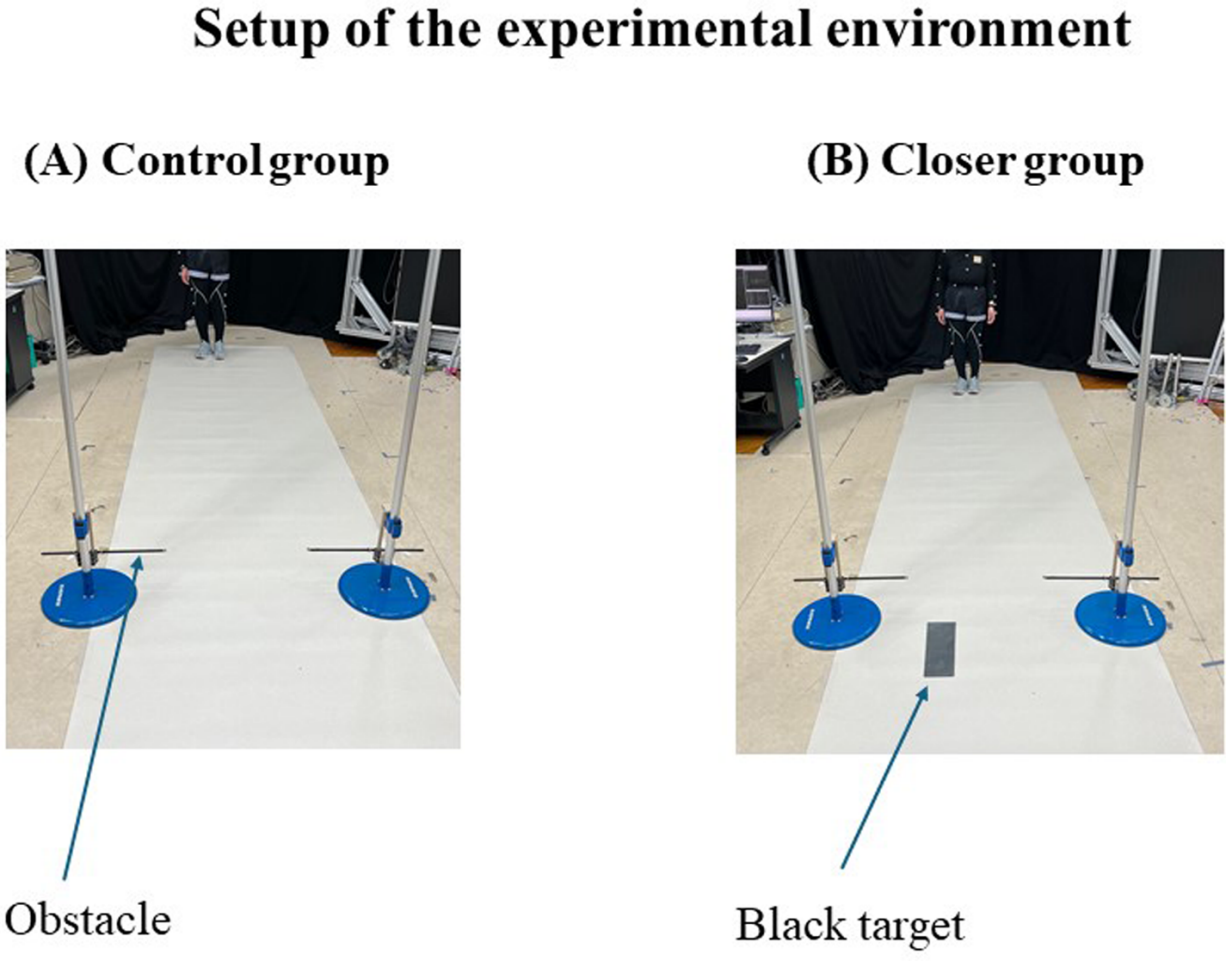
**(A)** Experimental setup for the control group, with two poles placed on either side with obstacles installed on both sides. In the control group, no target specified the placement of the leading limb (right foot), allowing participants to cross the obstacle naturally. **(B)** Experimental setup for the closer group, in which participants were instructed to place the leading limb (right limb) in the center of a marked position (black target) on the walking path after crossing the obstacle. The target was positioned at 0.5 times the leading limb placement distance measured for each participant in the pre-test. In both groups, obstacles were positioned outside the trajectories of the leading and trailing limbs, creating an experimental environment in which physical collisions and feedback from such collisions did not occur. Feedback on toe lift height was not provided in any pre-test, post-test, or during the intervention.

The experiment was conducted in a room measuring 6.6 m × 5.6 m (Figure 1A). Participants were instructed to walk a distance of 4 m from the starting line along a 5.5 m long walking path. An obstacle, consisting of a wooden board attached to two poles, was placed at a distance of 3 m from the start. The height of the obstacle was adjusted for each participant according to their lower-limb length. To prevent physical collisions, the wooden board was placed outside of the foot trajectory so that physical collisions between the obstacle and the foot did not occur. A black target square (30 × 10 cm) was placed on the walking path after the obstacle. Data collection was managed using a desktop computer (OMEN HP Obelisk Desktop 875-1xxx; HP, USA). Participants wore overhead headphones (Audio-Technica)) to block extraneous sound stimuli, with infrared reflective markers affixed to the headphones to track head position. A total of 42 reflective markers were used across the body and environment (see Appendix A for details). Eighteen cameras (OQUS and MIQUS, Qualisys, Sweden) were used to capture the spatial positions of the entire body, obstacle, and start and stop locations. Three-dimensional motion analysis was performed at a sampling frequency of 60 Hz. Data processing was performed using Visual 3D (version 6, C-Motion). The data were processed using a 4th-order Butterworth filter with a cutoff frequency of 4 Hz.

### Tasks and procedures

Participants were randomly assigned to one of the two intervention groups: closer (*n* = 11) or control (*n* = 10). The experiment consisted of four parts: (a) measurements of the participants, (b) baseline measurements of the step over an obstacle task (pre-test), (c) intervention, and (d) post-test measurements of the step over an obstacle task (post-test).

### Measurements of participant's details

The height and limb length of each participant were measured in centimeters. Limb length was measured as the distance from the greater trochanter to the plantar surface. Participants' cognitive and mobility functions were also measured. Cognitive function was evaluated using the MMSE, an 11-question test that assesses five cognitive domains, with a maximum score of 30 (22). Mobility function was assessed using the TUG test (23), in which participants were instructed to stand up after being seated on a standard chair with a seat height of 40 cm, walk 3 m at maximum speed, turn around, and return to sit in the chair. The time required from the verbal command to the initiation of sitting was recorded using a stopwatch. Each participant performed the TUG task twice, and the average of the two trials was calculated.

### Pre- and post-tests

The experimental task required participants to step over an obstacle placed 3 m from the starting position on the walking path. For baseline measurements, the participants stepped over obstacles naturally. The pole height was set at 20% of each participant's lower-limb length, defined as the distance from the greater trochanter to the plantar surface. This obstacle height has been widely used in previous studies (15, 26–28). At the start of each trial, participants stood at the designated starting position until prompted by the experimenter to begin. Each participant selected their initial step to initiate walking and then proceeded at a comfortable pace, stepping over the obstacle with the right limb and continuing to the end position. The obstacle was positioned outside the trajectories of the leading and trailing limbs to prevent collisions and thereby avoid any unintentional feedback that could influence avoidance behavior. To eliminate any influence of feedback on the experimental results, no feedback on toe lift height was provided during pre- and post-tests. No feedback on foot placement (i.e., black target) was provided to either group during the pre- and post-tests. In the subsequent intervention section, the procedures remained consistent, including the selection of the starting foot, maintaining a comfortable walking pace, positioning the obstacle outside of the foot trajectory, and not providing feedback on toe lift height. Participants completed a total of 10 main trials in both the pre- and post-test sessions. Prior to the main trials in the pre-test session, each participant performed three practice trials to familiarize themselves with the experimental procedure.

### Intervention

The intervention tasks for the closer and control groups are shown in Figures 1A,B. In the closer group, participants were instructed to place their leading limb (right limb) at the center of the marked position (black target) on the walking path after crossing the obstacle. The target position was set at 0.5 times the leading limb placement distance measured for each participant during the pre-test. This target placement was based on that used by Hakamata et al. (15), in which a similar placement reduced trailing limb collisions in younger individuals. Given that older adults typically exhibit greater variability in foot placement compared to younger adults (29), there was a concern that a uniform target position might not be an appropriate experimental manipulation for all participants. Therefore, we calculated the foot placement of the leading limb after crossing the obstacle, measured it in the pre-test, and determined the target position for each participant based on this value. This allowed us to address the variability in leading limb foot placement among participants and to perform sufficient experimental manipulation (0.5 times) for all participants in the closer group. In the control group, no target markers were used, and the participants were simply instructed to cross the obstacle with their right foot. Participants in each group completed 30 main trials, with three practice trials conducted beforehand to familiarize participants with the task.

### Data analyses

To test the homogeneity of participants between the two experimental groups, a *t*-test was applied to compare participants characteristics (i.e., age, height, length of the limb, MMSE score, and TUG test score), excluding gender. Pearson's chi-squared test was used to analyze the gender ratio between groups.

**Figure 2:**
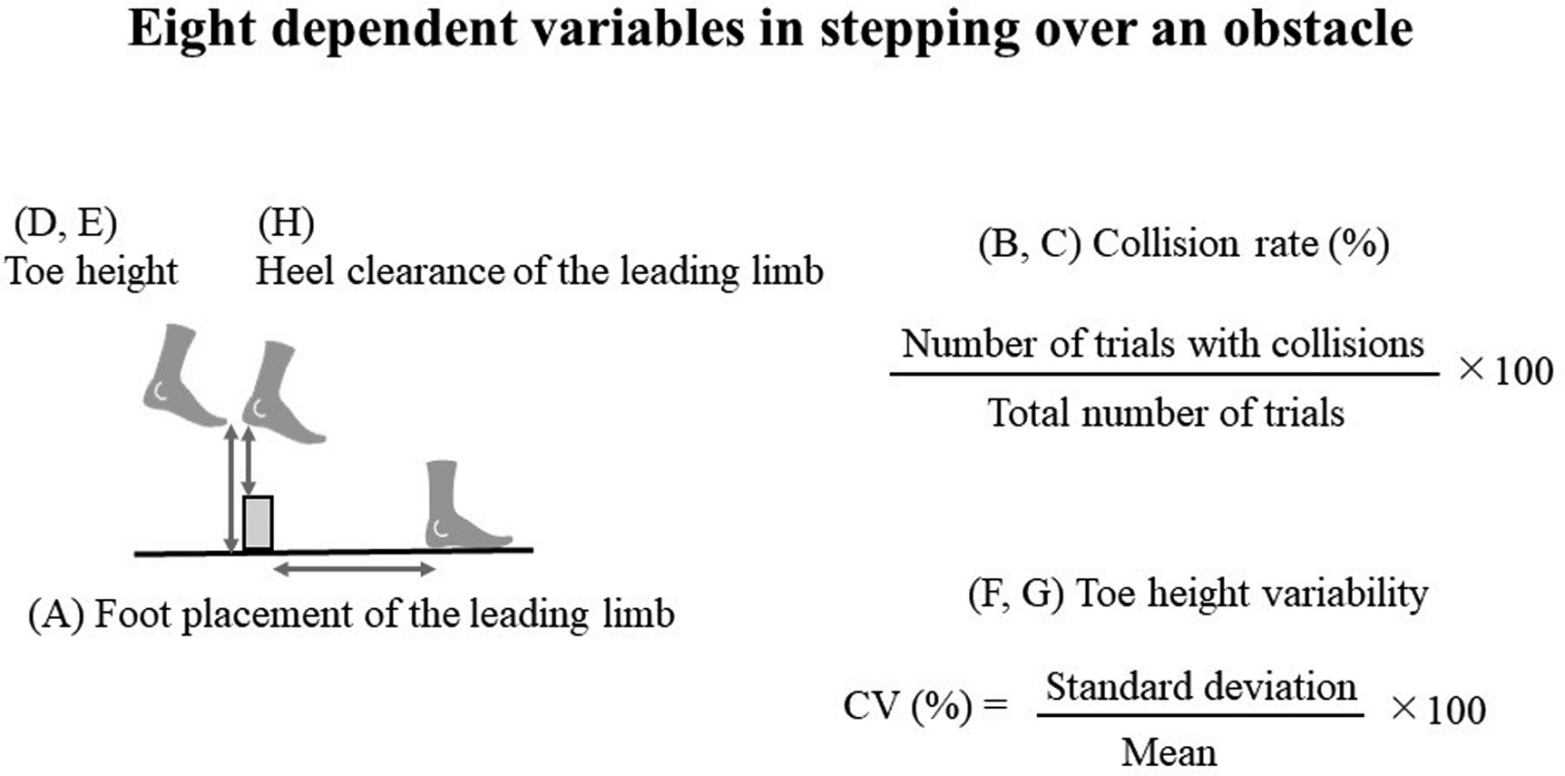
**(A)** Foot placement of the leading limb after stepping over the obstacle, **(B)** collision rate of the leading limb, **(C)** collision rate of the trailing limb, **(D)** toe height of the leading limb, **(E)** toe height of the trailing limb, **(F)** toe height variability of the leading limb, **(G)** toe height variability of the trailing limb, **(H)** heel clearance of the leading limb.

We measured the following six variables to address how participants stepped over an obstacle (see Figure 2): (A) foot placement of the leading limb after stepping over the obstacle, (B) collision rate of the leading limb, (C) collision rate of the trailing limb, (D) toe height of the leading limb, (E) toe height of the trailing limb, (F) toe height variability of the leading limb, (G) toe height variability of the trailing limb, (H) heel clearance of the leading limb, and (I) walking speed at obstacle crossing of the trailing limb. Notably, collisions of the leading limb did not occur during the pre- and post-tests. Therefore, leading limb collision rate was not statistically tested; we analyzed totally eight variables.

The first variable, foot placement after the leading limb crossed the obstacle, was defined as the horizontal distance between the heel marker of the leading limb and the obstacle. This distance was normalized to leg length to account for individual differences. Collision rates were calculated based on the vertical distance between the heel marker of the lead limb, the vertical distance between the second metatarsal markers of the lead and trailing limbs, and the position of the obstacle marker at the moment of crossing on the wooden board. Collisions were considered to have occurred when the vertical distance was less than zero. Toe height was defined as the point at which the leading and trailing limbs crossed the obstacle marker, and was measured as the vertical distance of the second metatarsal marker. Toe height was normalized to leg length to account for differences between participants. Toe height variability was calculated using the coefficient of variation (CV). The CV was calculated by dividing the standard deviation by the mean, and the result was multiplied by 100 and expressed as a percentage.

Heel clearance of the leading limb was defined as the point when the heel of the leading limb crossed the obstacle marker, and it was measured as the vertical distance between the heel marker of the leading limb and the obstacle. Heel clearance was normalized to leg length. Measurement of the heel clearance of the leading limb was necessary to confirm whether the intervention did not lead to the lower heel clearance. Considering that the heel of the leading limb is more likely to be in contact with the obstacle than the toe (30, 31), it is important to confirm that this intervention has not reduced heel clearance, making it more likely for the heel to collide with obstacles.

The walking speed of the trailing limb during obstacle crossing was calculated based on the anterior-posterior whole-body center of mass (COM) at the moment the trailing limb crossed the obstacle marker. The whole-body COM position was calculated as the sum of the body-segmented COM, with 38 reflective markers attached to the entire body (see Appendix A for detail of the location of these reflexive markers).

For each variable, a two-factor (group × test) repeated analysis of variance (ANOVA) was conducted. Because the distribution was not normal for the collision rate of the trailing limb, the data were adjusted using logit-transformation (32). When a significant interaction effect was found, Scheffe's *post-hoc* test was conducted. The level of significance for all analyses was set at *p* < 0.05.

## Results

Participant characteristics are summarized in Table 1. No significant differences were found between the two groups across any measurements.

**Table 1:** Participants characteristics.

	Closer group (*n* = 11)	Control group (*n* = 10)	*P* value
Gender(male/female)^a^	6/5	5/5	0.83
Age (year)^b^	74.7 ± 4.3	76.8 ± 7.0	0.41
Height (cm)^b^	160.5 ± 9.7	164.3 ± 11.8	0.42
Length of limb (cm)^b^	79.5 ± 3.7	81.0 ± 5.5	0.48
MMSE (points)^b^	29.9 ± 0.3	29.6 ± 0.7	0.19
TUG (s)^b^	7.2 ± 0.5	7.0 ± 0.6	0.28

MMSE, mini-mental state examination; TUG, timed up & go test.

^a^ Pearson's chi-square test.

^b^ Independent *t*-test.

**Figure 3:**
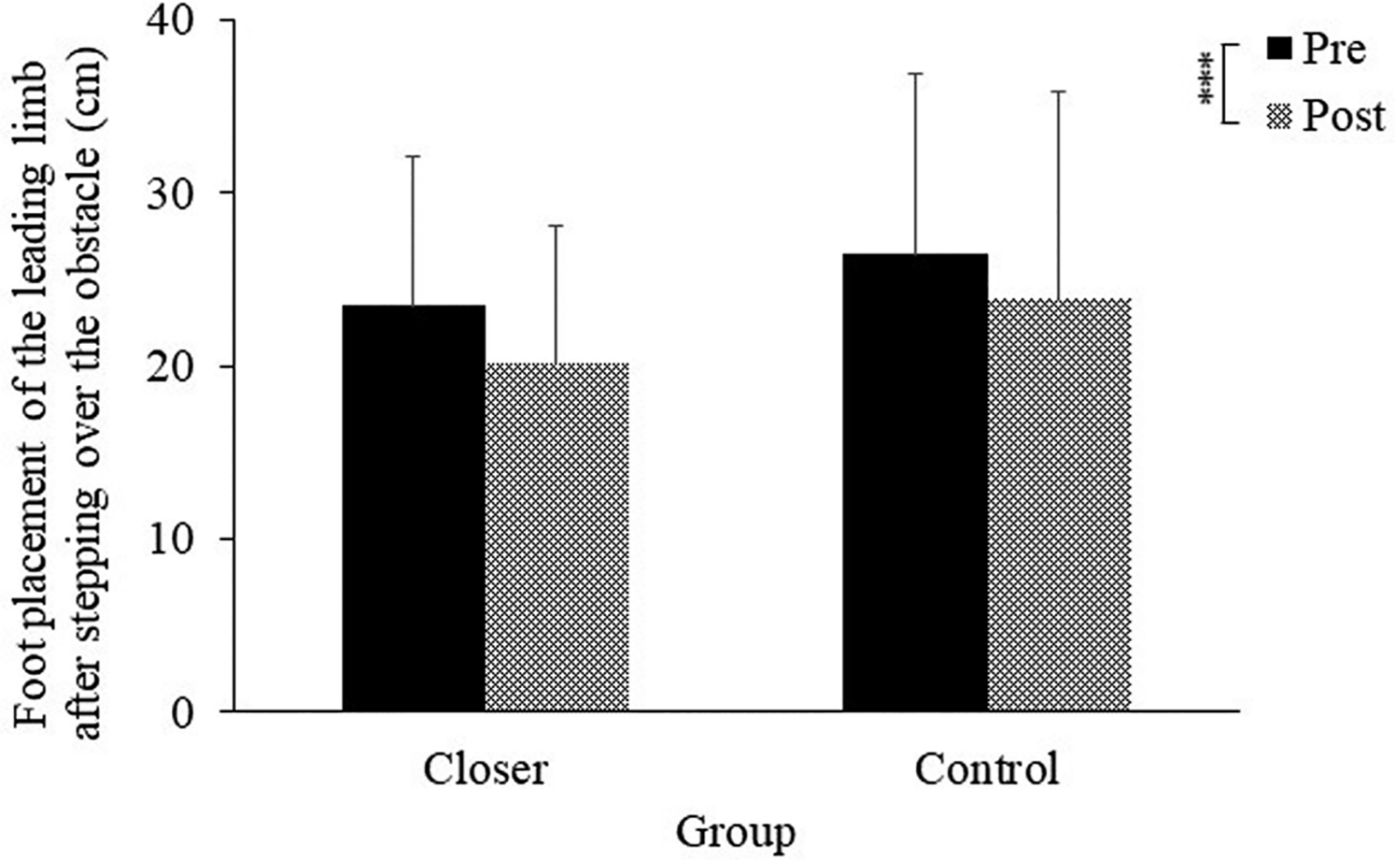
Foot placement of the leading limb after stepping over the obstacle. Significance levels are indicated by ***p < 0.001.

The mean foot placement of the leading limb after stepping over the obstacle in each experimental group is shown in Figure 3. The ANOVA showed no main effect of group [*F* (1, 19) = 0.63, *p* = 0.43, *η_p_^2^* = 0.03, *ns*]. However, a significant main effect of the test was observed [*F* (1, 19) = 21.81, *p* < 0.001, *η_p_^2^* = 0.53], indicating that both groups showed a significant change in the mean foot placement of the leading limb from pre-test to post-test. The interaction between the two factors was not significant [*F* (1, 19) = 0.38, *p* = 0.54, *η_p_^2^* = 0.01, *ns*].

**Figure 4:**
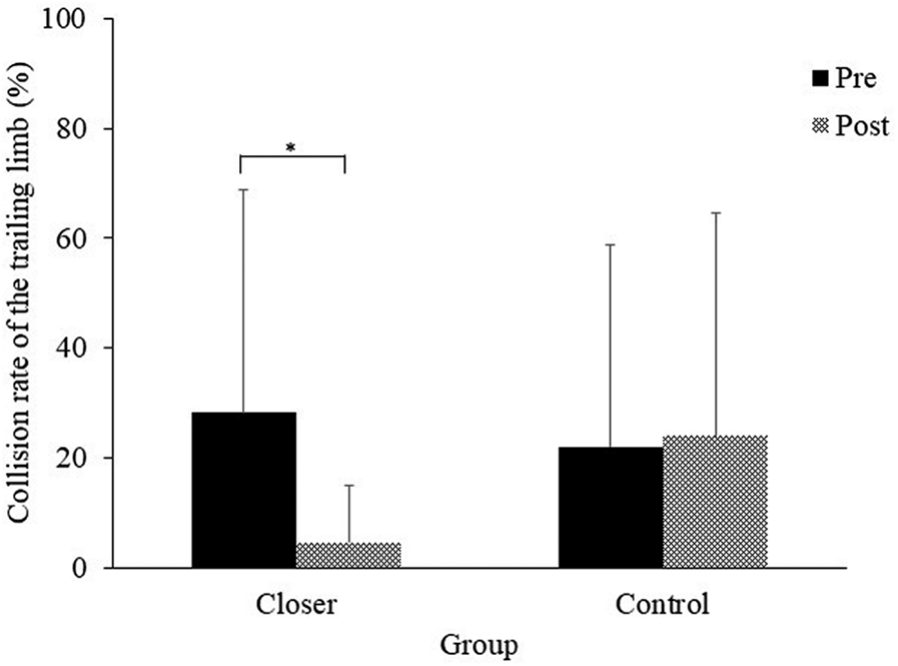
Collision rate of the trailing limb. Significance levels are indicated by *p < 0.05.

The mean collision rate of the trailing limb for each experimental group is shown in Figure 4. The ANOVA conducted on the logit -transformed collision rate data showed that neither the main effect of group [*F* (1, 19) = 0.34, *p* = 0.56, *η_p_^2^* = 0.01, *ns*] nor the main effect of the test [*F* (1, 19) = 0.75, *p* = 0.39, *η_p_^2^* = 0.03, *ns*] was significant. However, a significant interaction effect was observed [*F* (1, 19) = 7.08, *p* = 0.01, *η_p_^2^* = 0.27], with the collision rate in the closer group significantly lower in the post-test compared to the pre-test (*p* = 0.02).

**Figure 5:**
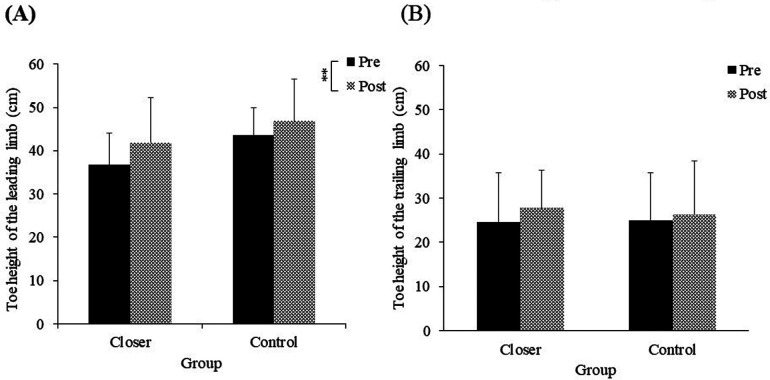
**(A)** Toe height of the leading limb, **(B)** Toe height of the trailing limb. Significance levels are indicated by **p < 0.01.

The mean toe height of the leading limb in each experimental group is shown in Figure 5A. The ANOVA showed no significant main effect of group [*F* (1, 19) = 2.83, *p* = 0.10, *η_p_^2^* = 0.12, *ns*]. However, a significant main effect of the test was observed [*F* (1, 19) = 9.81, *p* = 0.005, *η_p_^2^* = 0.34], indicating that both groups showed a significant change in the mean toe height of the leading limb from pre-test to post-test. Additionally, the interaction between the two factors was not significant [*F* (1, 19) = 0.48, *p* = 0.49, *η_p_^2^* = 0.02, *ns*].

The mean toe height of the trailing limb in each experimental group is shown in Figure 5B. The ANOVA showed no significant main effect of group [*F* (1, 19) = 0.01, *p* = 0.89, *η_p_^2^* = 0.00, *ns*] and no significant main effect of the test [*F* (1, 19) = 1.37, *p* = 0.25, *η_p_^2^* = 0.06, *ns*]. Additionally, the interaction between the two factors was not significant [*F* (1, 19) = 0.28, *p* = 0.59, *η_p_^2^* = 0.01, *ns*].

**Figure 6:**
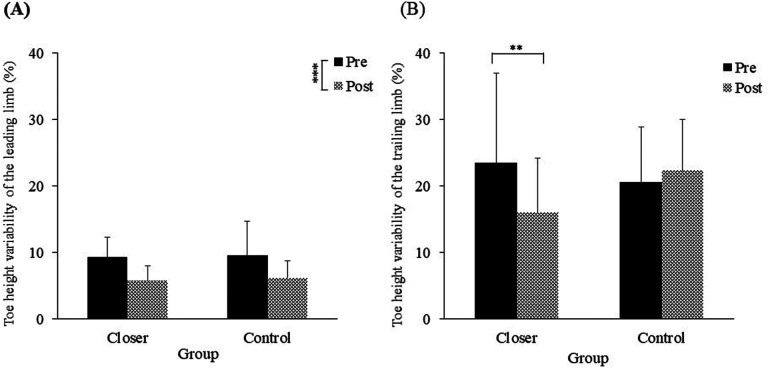
**(A)** Toe height variability of the leading limb, **(B)** Toe height variability of the trailing limb. Significance levels are indicated by **p < 0.01, ***p < 0.001.

The mean toe height variability of the leading limb in each experimental group is shown in Figure 6A. The ANOVA showed no significant main effect of group [*F* (1, 19) = 0.05, *p* = 0.81, *η_p_^2^* = 0.00, *ns*]. However, a significant main effect of the test was observed [*F* (1, 19) = 24.47, *p* < 0.001, *η_p_^2^* = 0.56], indicating that both groups showed a significant change in the mean toe height variability of the leading limb from pre-test to post-test. Additionally, the interaction between the two factors was not significant [*F* (1, 19) = 0.01, *p* = 0.89, *η_p_^2^* = 0.00, *ns*].

The mean toe height variability of the trailing limb in each experimental group is shown in Figure 6B. The ANOVA revealed no significant main effect of group [*F* (1, 19) = 0.18, *p* = 0.67, *η_p_^2^* = 0.00, *ns*] or test [*F* (1, 19) = 2.45, *p* = 0.13, *η_p_^2^* = 0.11, *ns*]. However, a significant interaction effect was observed [*F* (1, 19) = 6.41, *p* = 0.02, *η_p_^2^* = 0.25], with toe height variability in the closer group significantly lower in the post-test compared to the pre-test (*p* < 0.01).

**Figure 7:**
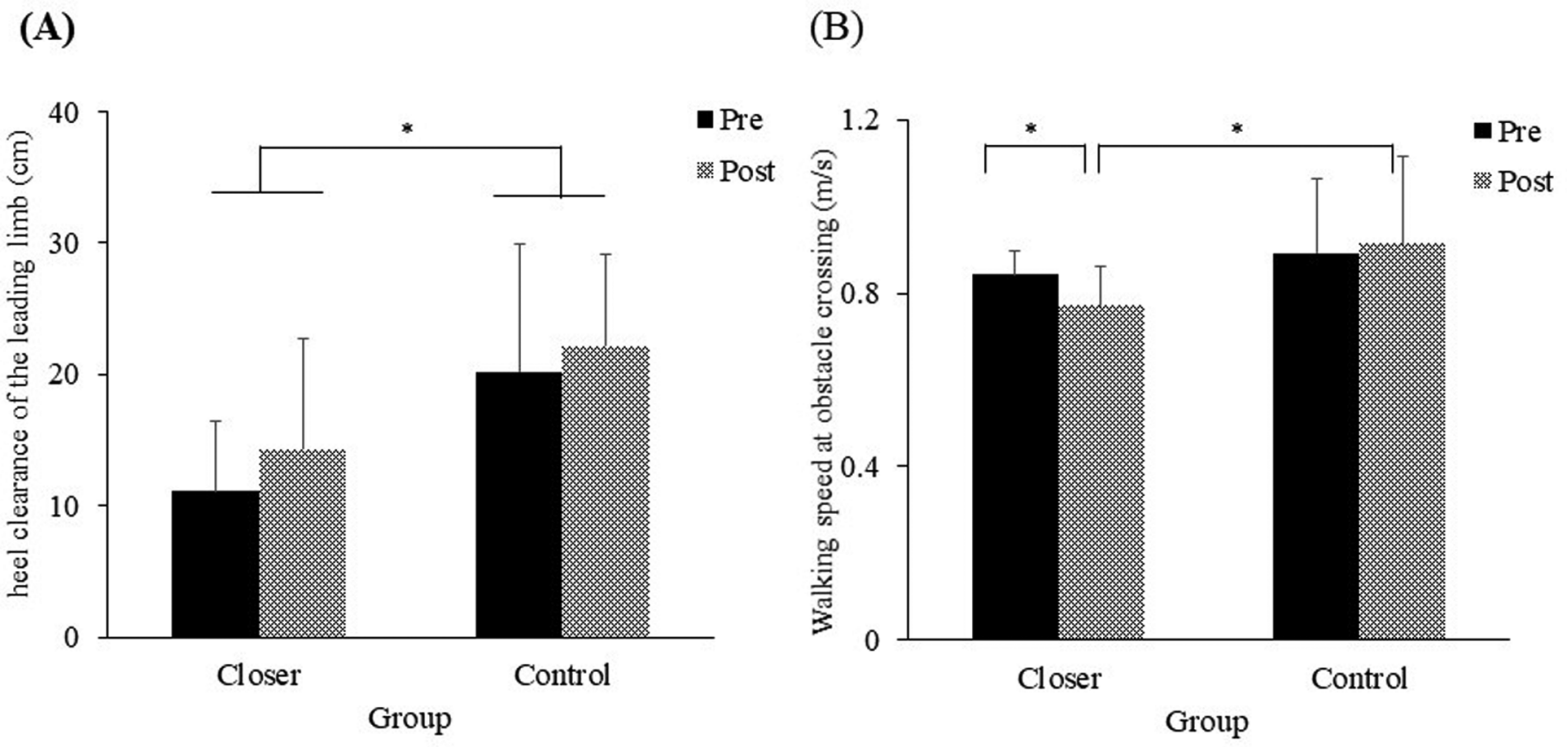
**(A)** Heel clearance of the leading limb, **(B)** walking speed at obstacle crossing of the trailing limb. Significance levels are indicated by *p < 0.05.

The mean heel clearance of the leading limb in each experimental group is shown in Figure 7A. The ANOVA revealed a significant main effect of group was observed [*F* (1, 19) = 7.66, *p* = 0.01, *η_p_^2^* = 0.28], indicating that the heel clearance of the leading limb differed between the groups. No significant main effect of test [*F* (1, 19) = 3.33, *p* = 0.08, *η_p_^2^* = 0.14, *ns*] or interaction was observed [*F* (1, 19) = 0.20, *p* = 0.65, *η_p_^2^* = 0.01, *ns*].

The mean walking speed at the obstacle crossing of the trailing limb in each experimental group is shown in Figure 7B. The ANOVA indicated no significant main effect of group [*F* (1, 19) = 2.88, *p* = 0.10, *η_p_^2^* = 0.13, *ns*] or test [*F* (1, 19) = 1.37, *p* = 0.25, *η_p_^2^* = 0.06, *ns*]. However, a significant interaction effect was observed [*F* (1, 19) = 4.56, *p* = 0.04, *η_p_^2^* = 0.19]. In the closer group, walking speed was significantly slower in the post-test compared to the pre-test (*p* = 0.02), and post-test walking speed was significantly slower in the closer group than in the control group (*p* = 0.04).

## Discussion

In this study, we examined whether an intervention to place the foot placement of the leading limb closer after crossing an obstacle would reduce collisions in the trailing limb of healthy older adults. As hypothesized, the results showed that the collision rate of the trailing limb in the closer group was significantly lower in the post-test than in the pre-test (Figure 4). Additionally, reductions in both toe height variability and walking speed for the trailing limb further supported this hypothesis (Figures 6B, 7B). Although toe height tended to be greater in the closer group (Figure 5B), this increase was not statistically significant, providing partial support for the second hypothesis related to careful controlled limb movement. These findings suggest that an intervention involving placing the leading limb closer to the obstacle effectively enhances collision avoidance for the trailing limb, even among healthy older adults.

When the collisions of the trailing limb were reduced, careful control was observed. Previous research has shown that when attentional demands increase during stepping tasks, individuals reduce toe height and decrease variability in foot lift for both the leading and trailing limbs to minimize the risk of tripping and associated injury (33). Similarly, in tasks requiring greater attentional load during walking, older adults have been shown to reduce foot placement variability, maintain a consistent foot height (34), and reduce walking speed (35). These modifications in gait are understood as strategies to enhance stability and reduce the likelihood of tripping or falling (34, 35). In other words, during tasks that require heightened attention, older adults may exhibit careful control by reducing foot placement variability and decreasing walking speed. Additionally, it has been noted that older adults tend to place their leading limb closer to obstacles after stepping over them compared to younger adults (29). Considering these previous studies, the present findings suggest that the intervention to place the leading limb closer to the obstacle encouraged participants to adopt strategies that reduce toe height variability and walking speed, thereby supporting tripping and falling prevention. Moreover, both groups showed an increase in the mean foot height and a decrease in the variability of toe height for the leading limb in the post-test, with the placement of the leading limb's foot becoming closer to the obstacle (Figures 3, 5A, 6A). These changes were similar in both groups, indicating that the repeated obstacle-crossing task might have influenced older adults.

Careful control facilitated safe obstacle avoidance, consistent with the speed-accuracy trade-off principle a foundational concept in human motor control (36, 37). Previous research has shown that trade-offs apply to obstacle avoidance (15, 38), walking (39), and stepping movements of the lower limbs (40). Patla et al. (41) noted that the vertical speed of the trailing limb exceeds that of the leading limb during obstacle crossing. The observed reduction in trailing limb collisions among younger participants in a VR environment has been attributed to a slower anterior-posterior passing speed (15). Moreover, increased variability in obstacle clearance can elevate the risk of contact, potentially increasing the risk of tripping and falling (42). Based on these previous studies, the findings of this study suggest that strategies aimed at reducing toe height variability and slowing passing speed were employed when trailing limb collisions were reduced.

Placing the leading limb closer to an obstacle is generally considered to increase collision risk (43). However, careful control collision avoidance is more feasible for the leading limb than the trailing limb due to the availability of visual, particularly peripheral, input for controlling the leading limb's movements (18, 44). In this study, no collisions were observed for the leading limb. By placing the leading limb closer to the obstacle, conditions were established that reduced the likelihood for trailing limb collisions. Previous studies have proposed the possibility of an interaction between the leading and trailing limbs (45) and indicated that certain visual-cognitive processes involved in obstacle crossing are shared between the two limbs (46). Additionally, it has been suggested that the trailing limb may use proprioceptive information from the leading limb during crossing movements (47). Based on these previous studies, this study leveraged visual information from the leading limb to enable foot placement closer to the obstacle after crossing without incurring collisions. The trailing limb may reduce collision risk by utilizing shared information from the leading limb during the crossing movement.

One may assume that the intervention in the current study may increase the risk of the contact with the heel of the leading limb. Previous studies have indicated that the heel of the leading limb is more likely to be in contact with the obstacle than the toe (30, 31). Given that that recovering from a lead limb trip is more challenging than recovering from a trail limb trip and that the leading limb is more prone to collisions during the late swing phase (48), placing the heel of the leading limb too close to an obstacle after stepping over it is not advisable. To avoid the risk, it was necessary to appropriately place the marked position to be stepped on, ensuring enough space to avoid a collision, even if it meant landing closer to the obstacle than usual. The results of the current study showed that (a) the mean foot placement of the leading limb after stepping over the obstacle was approximately 20 cm in the post-test for the closer group (Figure 3) and (b) no collision with the leading limb occurred. Additionally, we confirmed that the heel clearance of the leading limb was sufficient to avoid colliding with the obstacle (Figure 7A). Although a difference in heel clearance was observed between the two groups, in the closer group, the value of heel clearance did not decrease significantly after the intervention, suggesting that the intervention did not reduce heel clearance. In fact, no collisions occurred in any of the trials. Taken collectively, these findings suggest that our setting did not increase the risk of contact with the heel of the leading limb. In other words, this study's intervention demonstrated a reduction in trailing limb collisions without causing leading limb collisions.

In this research, the collision rate during the pre-test was approximately 25%, which is higher than the 0.6% reported in a previous study (16). We consider that this relatively higher collision rate is primarily attributed to the experimental setup, where collision feedback was absent, making it difficult for participants to notice collisions. In support of this interpretation, Heijnen et al. reported that collision rates increase to approximately 47% when collision feedback is absent and obstacles are invisible (17). Additionally, age-related changes in the use of sensory input to monitor the movement of the trailing limb may have been involved. Previous research has indicated that older adults tend to rely more on their central vision than younger individuals while walking (49). Because the obstacle is out of sight when stepping over it with the trailing limb, older adults may have found it difficult to accurately monitor the movement of the trailing limb in situations where no collision feedback was provided.

This study has several limitations. Firstly, although the required number of participants was met according to the *a priori* power analysis, it cannot be ruled out that the unequal sample sizes between the closer group and the control group may have influenced the results (i.e., an increased risk of Type I errors). Second, the effectiveness of the intervention may be specific to a group of healthy older adults. Future research should examine whether the collision reduction effects extend to individuals with a history of falls or under conditions such as fractures. Third, the generalizability of results from an experimental setting in which physical collisions do not occur to everyday situations remains unclear. For example, a previous study reported a collision rate of 1.6% in older adults (50), whereas the average collision rate in both groups in the pre-test in this study was 25%. Therefore, further studies are required to confirm whether the experimental tasks employed here translate to real-life scenarios. Fourthly, this study only assessed the immediate effects of the intervention on leading limb placement. Similar to previous research (51), future studies should investigate the retention effects of this intervention over time.

In conclusion, this study demonstrated that an intervention to place the foot placement of the leading limb closer to the obstacle reduces collision avoidance by the trailing limb in older adults. This indicates that careful control was observed, as evidenced by the reduction in toe height variability and decreased passing speed of the trailing limb. Future research should investigate whether such interventions can further improve collision avoidance, particularly in populations more vulnerable to collisions, such as older adults with post-fall conditions.

## Data Availability

The raw data supporting the conclusions of this article will be made available by the authors, without undue reservation.

## Appendix A

A total of 42 reflective markers were placed on the body and in the environment. For each participant, 38 markers were attached to specific sites on the body: three markers on the headphones, seven on the trunk (superior end of the sternum, xiphoid process, seventh cervical vertebra, 10th thoracic vertebra, right and left acromion, and right scapula); 12 markers were attached to the right and left upper extremities (six locations on each arm: humerus, lateral epicondyle of the humerus, dorsal forearm, medial and lateral wrist joints, and dorsal third finger); four on the pelvis (left and right superior anterior iliac spines and left and right superior posterior iliac spines); and 12 markers on the lower extremities (six locations on each leg: lateral femur, lateral femoral epicondyle, lateral lower limb, external ankle joint, second metatarsal bone, and upper calcaneus bone). Additional reflective markers were placed in the environment for motion analysis: one marker on each vertical pole indicating the obstacle location, as well as markers at the start and stop positions.
